# High-Resolution Measurement of Surface Normal Maps Using Specular Reflection Imaging

**DOI:** 10.3390/jimaging12040164

**Published:** 2026-04-10

**Authors:** Shinichi Inoue, Yoshinori Igarashi, Seiji Suzuki

**Affiliations:** 1 Faculty of Engineering, Tokyo Polytechnic University, 5-45-1 Iiyamaminami, Atsugi 243-0297, Japan; 2 CHUO PRECISION INDUSTRIAL CO., LTD., 65 Shirasaka Miwadai, Shirakawashi 961-0835, Japan; igarashi@chuo.co.jp (Y.I.); s-suzuki@chuo.co.jp (S.S.)

**Keywords:** surface normal vectors, normal maps, measurement method, specular reflection, appearance, surface analysis, industrial inspection

## Abstract

This paper presents a method for measuring the spatial distribution of surface normal vectors with high angular accuracy. The measured data are visualized using a color-mapping technique and represented as normal maps, which are commonly used in computer graphics. Reliable methods for evaluating material surface properties have long been sought in industrial applications where visual assessments of reflective properties are still widely employed, particularly in appearance-critical fields. Motivated by this need, we introduce an imaging-based technique for measuring the high-resolution spatial distribution of surface normal vectors from specular reflection. A dedicated measurement apparatus was developed to capture surface normal vectors at 1024 × 1024 sampling points with a spatial resolution of 0.02 × 0.02 mm and an angular resolution of approximately 0.1°. Using this apparatus, normal maps were obtained for various materials, including plastic, ceramic tile, inkjet paper, and aluminum sheets. The spatial distribution of surface normal vectors reflects surface roughness, which strongly influences perceived texture. The resulting normal maps enable not only quantitative surface analysis for industrial inspection but also the physical reproduction of gloss in computer graphics.

## 1. Introduction

In industrial sectors, the evaluation of material surface properties is a critical process that strongly influences product performance and reliability. In particular, measurements of height distribution and surface roughness associated with surface topography are widely conducted. On the other hand, when materials are used as final products, surface properties directly affect the appearance perceived by human users [1,2,3,4,5,6]. Fine surface asperities modify visual impressions such as gloss through their influence on the reflection and scattering of light. Although the appearance of a material can be recorded using photographs, such records represent only a specific phenomenon under a particular illumination condition. Consequently, there is a growing need for methodologies that can quantitatively capture these phenomena from a physical perspective.

This paper presents a method for measuring the spatial distribution of surface normal vectors with high angular accuracy. By adopting an approach that accounts for imaging technology, we aim to measure and record the physical properties of materials in a manner that is independent of specific illumination and observation conditions.

Visual evaluation of reflective properties is still widely performed, particularly in fields where appearance plays a critical role [3,4,5,6]. Motivated by this practice, we propose measuring the high-resolution distribution of surface normals based on reflective properties. A macroscopic surface can be regarded as an assembly of mesoscopic facet surfaces, and the spatial distribution of their normal vectors reflects surface roughness. This distribution gives rise to reflection irregularities and significantly influences perceived texture. In computer graphics such a distribution is commonly represented by a normal map. Because normal maps are stored as arrays of normal vectors.

**Figure 1:**
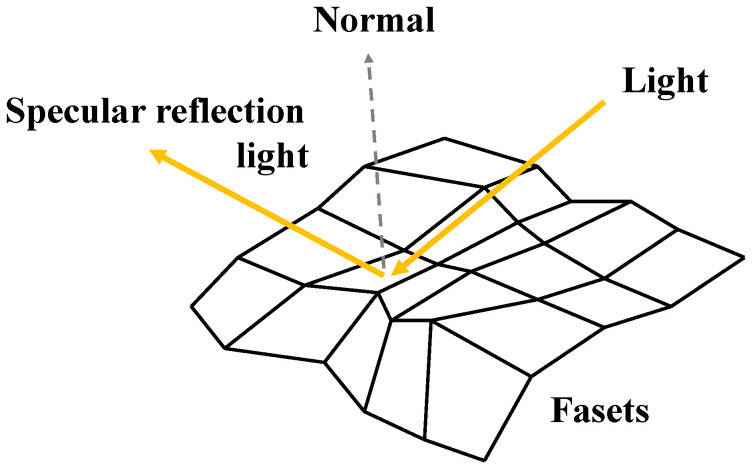
Schematic diagram of a macroscopic surface composed of mesoscopic facets. The direction of specular reflection depends on the orientation of the surface normals.

**Figure 2:**
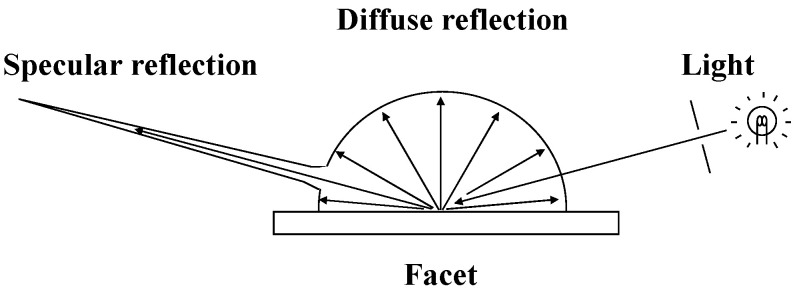
Schematic diagram of the dichromatic reflection model. The reflectance is described by the BRDF, which defines the angular distribution of reflected light.

The distribution of surface facets and their normals is illustrated in Figure 1. In three-dimensional space, a surface normal is defined as a vector perpendicular to the corresponding local tangent facet. The reflection light on each facet varies depending on the normal vector. The intensity of the reflected light is greatest under specular reflection conditions. The distribution of reflected light is illustrated in Figure 2. This distribution of reflectance intensity is described by the bidirectional reflectance distribution function (BRDF), which defines how light from a source is reflected by a surface. In the dichromatic reflection model, the reflected light intensity is expressed as the sum of diffuse and specular components. As shown in Figure 2, part of the incident light is absorbed, scattered, and reflected in many directions; this is referred to as diffuse reflection. Printed images are primarily observed through diffuse reflection. Specular reflection, in contrast, is mirror-like and highly directional. The physical basis of gloss is specular reflection.

Variations in surface normals lead to changes in specular reflection near the specular direction. The corresponding reflectance distribution typically exhibits a convex profile with a peak at this direction, where the reflected intensity is maximized.

**Figure 3:**
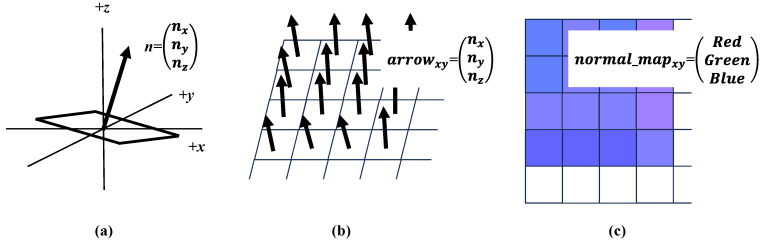
Schematic diagram illustrating a method for visualizing the spatial distribution of surface normal vectors. (**a**) Normal vector of a facet. (**b**) Arrow-based representation of the surface normal distribution. (**c**) Color-map representation of the surface normal distribution. A normal map is one type of color map commonly used in computer graphics. Here, **n** denotes the surface normal vector.

To visualize the measured data, a color-mapping technique is applied, and the results are represented as normal maps commonly used in computer graphics. A normal map is a record of normal vectors by encoding their components into three color channels, as shown in Figure 3. The spatial distribution of normal vectors can be displayed as a color image, making it easier to visually grasp surface characteristics. In addition, normal maps can be used to reproduce surface appearance using computer graphics.

Various methods based on deflectometry have been investigated as a means of directly measuring the spatial distribution of surface normal vectors [7]. Methods using photometric stereo and AI-based estimation have also been proposed to obtain depth maps [8,9]. Other approaches measure the spatial distribution of surface height and derive surface normals from its gradients. Stylus-type profilometers, white-light interferometers, and confocal microscopes are commonly used to measure high-resolution surface structures [10,11,12]. However, when the objective of measuring the normal distribution is to evaluate the appearance of specular reflection, these techniques provide only indirect measurements. Methods for measuring the normal vectors of a single surface point using reflectance are well established. The reflectance of an object’s surface under varying incident light angles can be measured using a technique known as gonio-reflectance [13,14]. However, such measurements are generally limited to a single point rather than a spatial distribution, and they require scanning the surface using complex mechanisms and a considerable amount of measurement time [14,15].

The purpose of measuring the surface normal distribution in this study is to analyze the appearance of specular reflection. Therefore, the measurement specifications should be comparable to those of the human visual system. Based on the standard observer with a 2-degree field of view (CIE), the measurement area should be larger than 17 × 17 mm. Considering the spatial resolution of the human eye, the measurement resolution should be 0.1 mm or finer. The required angular range depends on the target material; however, to accommodate a wide range of materials exhibiting specular reflection, a range of approximately ±3.0° is required, whereas for highly glossy materials, a narrower range of approximately ±1.0° is sufficient [4,5,16]. The angular resolution must be sufficient to characterize the angular distribution within this range, and a resolution finer than 0.2° is desirable. When these specifications were adopted, no existing measurement instrument was found to satisfy all of them.

Consequently, a method for directly measuring the spatial distribution of surface normal vectors has long been sought. In this paper, we introduce a method for measuring high-resolution normal maps that represent the spatial distribution of surface normal vectors. We develop an optical system equipped with variable-direction incident light sources. This system illuminates the surface from multiple directions, captures images of specular reflection, and estimates surface normal vectors based on these images. Finally, we discuss potential industrial applications of the proposed method, as well as its applicability to computer graphics.

## 2. Methods

This section explains the proposed method from a theoretical perspective and presents the specifications of the apparatus implemented in practice. The apparatus specifications and experimental conditions are consistent with those used in the experiments.

### 2.1. Coordinate System and Notation

In this paper, the target surface is regarded as being composed of small planar facets, each associated with a surface normal. Two-dimensional positions on the target surface are denoted by *x* and *y*, while the direction perpendicular to the surface is denoted by *z*. The axes are defined according to a right-handed Cartesian coordinate system. The surface normal vector **n** is represented as a three-dimensional vector in the Cartesian coordinate system, as shown in Equation (1). In this study, we use the unit surface normal vector.

(1) $$n=\left(n_{x},n_{y},n_{z}\right),$$

The illumination direction, represented by the light vector *l*, and the direction of reflected light, represented by the specular reflection vector *r*, are also defined in the same coordinate system.

### 2.2. Proposed Method for Measuring Normal Vectors

Our method estimates normal maps by analyzing reflection images captured under multiple lighting conditions. This approach is based on the hypothesis that variations in reflection arise from surface irregularities, that is, mesoscopic surface features. The main challenge is to measure the entire surface normal map simultaneously.

The surface normal vector is estimated as follows. According to reflection theory, the specular reflection vector is determined by the incident light vector and the surface normal vector of a facet (see Figure 1). When the direction of the incident light vector is varied and the peak reflected light is assumed to correspond to specular reflection, the surface normal vector ***n*** can be estimated as the half-vector ***h*** between the incident light vector ***l*** and the specular reflection vector ***r***, as shown in Equation (2).

(2) $$n\;=\;h\;=\frac{l+r}{\left|l+r\right|},$$

We focus on two key techniques in the development of the measurement apparatus: variable-directional illumination using a collimated lighting system, and single-direction imaging using a telecentric optical system.

**Figure 4:**
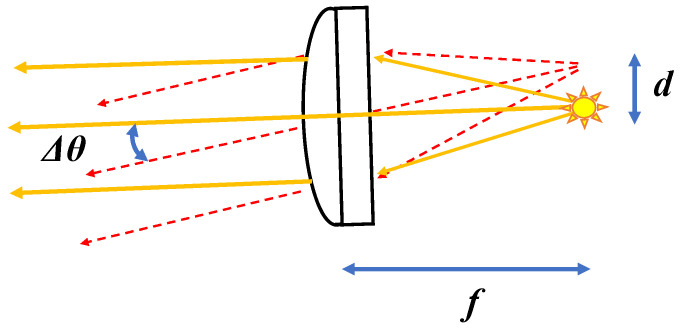
Schematic diagram of the collimator optical system. The distance from the optical axis, *d*, is determined by the focal length, *f*, and the illumination angle, Δ*θ*.

A schematic diagram of the variable-directional illumination system based on collimated lighting is shown in Figure 4. In collimator optics, a focal point exists on one side of a lens, while parallel light emerges on the other side. When a light source is placed at the focal point, the illumination system emits parallel (collimated) light.

The distance from the optical axis, *d*, is calculated from the illumination angle, Δ*θ*, and the focal length, *f*, as follows:

(3) $$d=f\cdot \tan\left(\Delta \theta \right),$$

In a conventional configuration, the light source is positioned at the focal point on the optical axis. In the newly developed apparatus, as shown in Figure 4, the light source position can be varied laterally to project collimated light at a desired illumination angle. As a result, the incident light angle is spatially uniform over the entire sample surface.

A variable-directional illumination system was developed using a lens system with a diameter of 46 mm and a focal length of 150.0 mm. The light source was a white LED with a 0.8 mm aperture mounted on an xy-stage. The minimum incremental motion of the xy-stage was 0.002 mm, corresponding to an angular change of 0.00076°.

**Figure 5:**
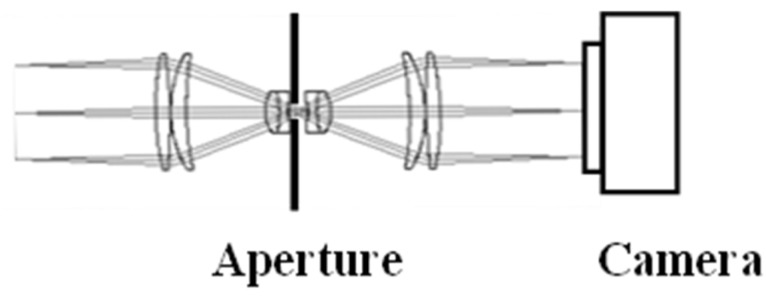
Schematic diagram of the telecentric optical system. The observation (measurement) angle is spatially uniform across the entire field of view.

To ensure that the observation angle is also spatially uniform over the entire sample, a telecentric optical system is employed. A telecentric optical system allows only light rays parallel to the optical axis to pass through the imaging optics, as illustrated in Figure 5.

The telecentric optical system employed was an Edmund Optics 0.30× SilverTL Telecentric Lens (Barrington, NJ, USA) with a lens diameter of 46 mm, a magnification of 0.30×, a numerical aperture (object side) of 0.025, and an f-number of 22.

**Figure 6:**
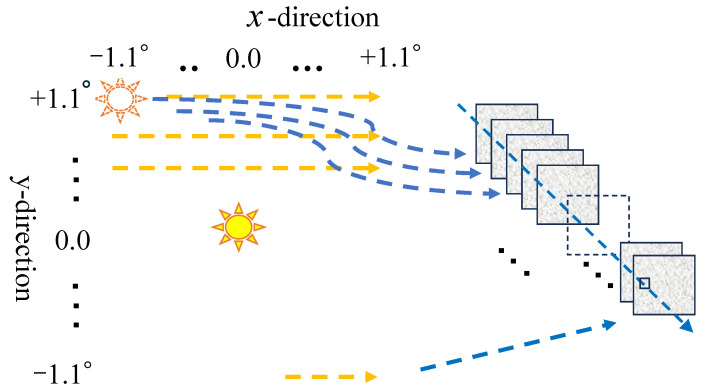
Schematic diagram of the processing procedure for estimating surface normal vectors. The incident light angle is varied continuously, and reflected images are captured at each angle. For each pixel, the incident angle that produces the maximum reflected intensity is identified, and the corresponding surface normal vector is estimated.

The processing procedure for estimating surface normal vectors is illustrated in Figure 6. The incident light angle is varied sequentially, and reflected images are captured at each illumination angle. For each pixel, the incident angle that maximizes the reflected intensity is then identified. Under this condition, the surface normal vector at each pixel is calculated using Equation (2). This procedure enables the surface normal map to be measured simultaneously over the entire field of view.

### 2.3. Proposed Method for Making Normal Maps

The proposed apparatus measures numerical surface normal data, (*n**_x_***, *n**_y_***, *n**_z_***), at each surface position, (*x*, *y*). These numerical values are essential for quantitative surface analysis. To reveal the overall surface characteristics, data visualization is applied. In this study, a color-mapping technique is employed to visualize the measured normal data and to facilitate intuitive understanding of the material surface properties.

The term normal map used in this paper is fundamentally identical to that used in the field of computer graphics. Normal mapping is a texture-mapping technique widely employed in computer graphics. A normal map is typically stored as an image in which the red, R, green, G, and blue, B, color channels correspond to the *n**_x_***, *n**_y_***, and *n**_z_*** components of the surface normal vector, respectively. The PNG file format is commonly used for this purpose. The R, G, and B values encode the *n**_x_***, *n**_y_***, and *n**_z_*** components of the unit normal vector, whose range from −1.0 to 1.0 is quantized into 256 intensity levels, as described below:

(4) $$R=255\cdot m_{x}\cdot (n_{x}+1)/2,$$

(5) $$G=255\cdot m_{y}\cdot (n_{y}+1)/2,$$

(6) $$B=255\cdot m_{z}\cdot (n_{z}+1)/2,$$

Here, *m_x_*, *m_y_* and *m**_z_*** denote scaling factors. The introduction of these scaling factors is an extension proposed in this study.

Normal maps that are currently widely used in computer graphics generally have a significantly lower angular resolution than that targeted in this study. To achieve higher angular resolution, we adopt a quantization scheme with scaled normal components. This approach increases angular resolution by effectively expanding the quantization levels. As a trade-off, the range of unit normal vectors that can be represented is limited. However, the variation in surface normal angles associated with specular reflection—which is the focus of this study—is relatively small. In this study, the scaling factor of ×32 was applied to the x- and y-components, with *m_x_* = *m_y_* = 32. The z-component of the unit normal vector is left unscaled, using a scaling factor of ×1, that is, *m_z_* = 1.

Handling the normal map as an image file in PNG format provides clear advantages for visualization. The PNG image representation of the normal map itself serves as a color-mapped visualization of the surface normal distribution. In the proposed method, inherently small variations in surface normal vectors are amplified for visualization purposes, making the surface condition easier to interpret. In this paper, such a visualization is referred to as a normal map (×32) and is presented as an enhanced visualization image of the measured normal map.

A trade-off arises in the choice of the scaling factor. With a scaling factor of 1, the angular range from −90.0° to +90.0° (corresponding to −1.0 to +1.0) on each axis is quantized into 256 levels. In this case, the quantization step is approximately 0.448° near the zenith, which is insufficient to represent a measurement resolution of 0.1°. By applying a scaling factor of 32, the quantization step is reduced to approximately 0.014° near the zenith, enabling higher-resolution representation. However, this reduces the representable angular range to approximately ±90.0°/32 (±2.8°). The prototype developed in this study handles data within ±1.1°. A scaling factor of 32 was selected to ensure that surface normals remain comparable even if the measurement angle range is expanded in the future.

**Figure 7:**
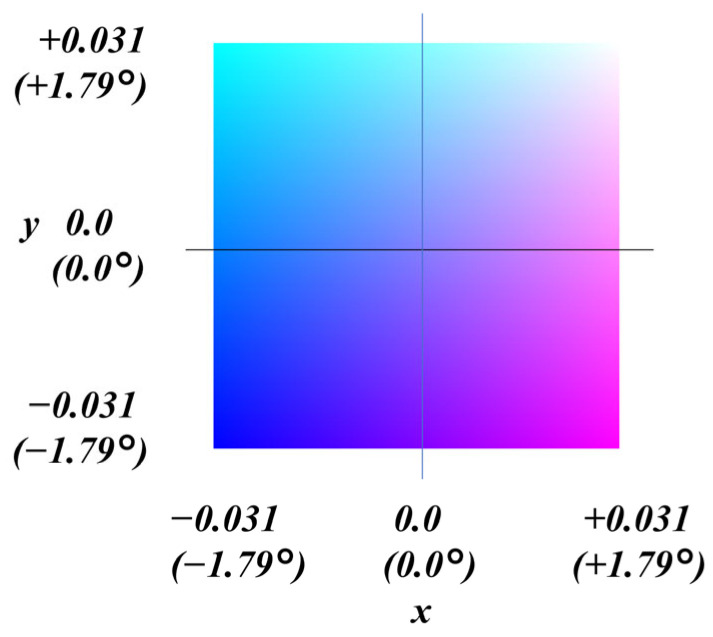
Color representation of surface normal vector directions in the proposed ×32-scaled normal map used for visualization.

In this normal map visualization, the direction of each unit normal vector is encoded as a color, as illustrated in Figure 7. This color representation should be used as a reference when interpreting the measurement results presented in this paper.

## 3. Experiment and Results

A measurement apparatus was developed for the proposed method. This apparatus is capable of measuring surface normal vectors at 1024 × 1024 sampling points with a spatial sampling interval of 0.02 × 0.02 mm, achieving an angular resolution of approximately 0.1°. Using this apparatus, normal maps were obtained for various materials, including plastic, ceramic tile, inkjet paper, and aluminum sheets.

### 3.1. Developed Measurement Apparatus

**Figure 8:**
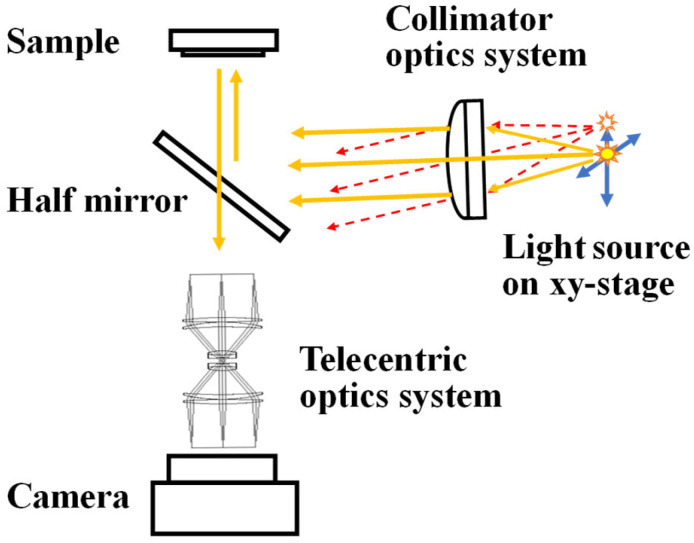
Schematic diagram of the measurement apparatus equipped with a variable-directional illumination system.

**Figure 9:**
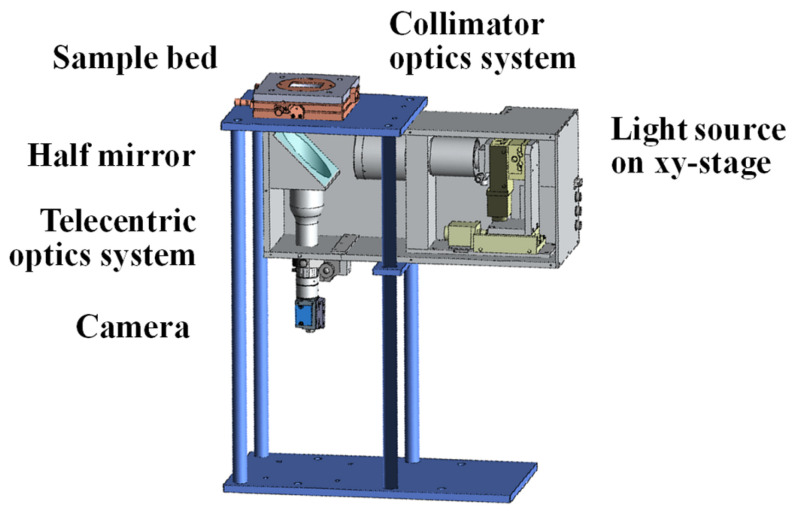
Computer graphics rendering of the measurement apparatus equipped with a variable-directional illumination system.

The developed measurement apparatus is shown in Figure 8 and Figure 9. Collimated light emitted from a collimator lens is reflected by a half mirror and directed onto the sample surface. The angle of the CMOS camera (DFK 33UX249, The Imaging Source, made in Bremen, Germany) is fixed at 0° relative to the sample surface. The effective measurement area is 20.48 × 20.48 mm, corresponding to an image resolution of 1024 × 1024 pixels.

The sample material is placed on a sample stage, and image acquisition is performed in a darkroom environment. The apparatus was developed by Chuo Precision Industrial Co., Ltd. (Tokyo, Japan), made in Japan.

### 3.2. Estimation of Surface Normal Vectors

Surface normal vectors are estimated as follows. The apparatus captures reflected images under multiple illumination angles, as illustrated in Figure 6. The observation vector ***v*** is fixed and perpendicular to the sample surface. For each pixel, the incident light vector corresponding to the maximum reflected light intensity is assumed to satisfy the specular reflection condition. Under this condition, the observation vector ***v*** coincides with the specular reflection vector ***r*** defined in Equation (2). Consequently, the surface normal vector ***n*** is given by the half-vector ***h*** between the incident light vector ***l*** and the reflection vector ***r***, as expressed in Equation (2).

The incident light angle is varied ±11 steps at intervals of 0.2° along both the x- and y-axes. As a result, a total of 529 reflected images are captured. Because the surface normal vector is estimated as a half-vector, the measurable angular range of the surface normal is ±1.1° along both axes, with an angular resolution 0.1°.

Approximately 6 min are required to capture 529 images, and an additional 4 min are needed to process the data and compute the normal map. Due to an opening in the housing of the prototype, measurements must be conducted in a completely dark environment. The system is therefore intended for offline use.

### 3.3. Measurement Results

**Figure 10:**
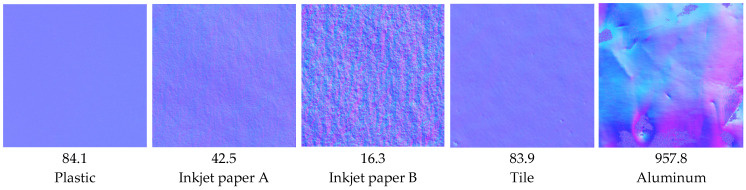
Normal maps obtained from the measurements for various materials. The middle row shows the ISO 20° specular gloss values, *Gs* (20°).

Surface normal vectors were measured and corresponding normal maps were generated for various materials. The measurement results are presented in Figure 10.

The samples included plastic (acrylate), a ceramic tile, inkjet paper A and inkjet paper B (photographic glossy types from different manufacturers), and an aluminum sheet. All samples had flat surfaces and were white, except for the aluminum sample, which had a silver appearance, and all exhibited mirror-like gloss. For reference, the ISO 20° specular gloss values (GU) of the samples are also shown. These measurements were performed using a RHOPOINT IQ Flex 20 gloss meter Rhopoint Instruments, made in St. Leonards-on-Sea, UK).

### 3.4. Verification of Measurement Accuracy

**Figure 11:**
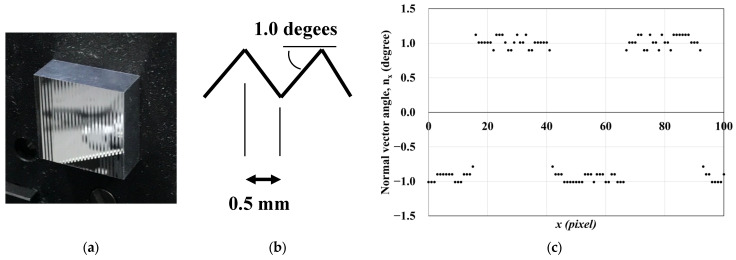
Verification of measurement accuracy I: calibration sample. Surface normal vectors measured at each position along the x-axis using the proposed measurement apparatus. (**a**) Photograph of the aluminum calibration sample. (**b**) Schematic diagram of the calibration sample geometry. (**c**) Measured surface normal vector distribution along the x-axis.

The accuracy of the proposed measurement apparatus was verified using a calibration sample, as shown in Figure 11. The calibration sample was fabricated from aluminum by Toray Precision Inc. (Otsu-shi, Japan). It consisted of a double-striped surface structure with a pitch of 1.0 mm (stripe width of 0.5 mm) and surface tilt angles of +1.0° and −1.0°. The surface exhibited mirror-like specular reflection. The measured normal vector values showed good agreement with the analytically calculated results. In particular, the measured spacing between adjacent extrema corresponded to 0.5 mm at 25 measurement points, which is consistent with the designed geometry of the calibration sample.

**Figure 12:**
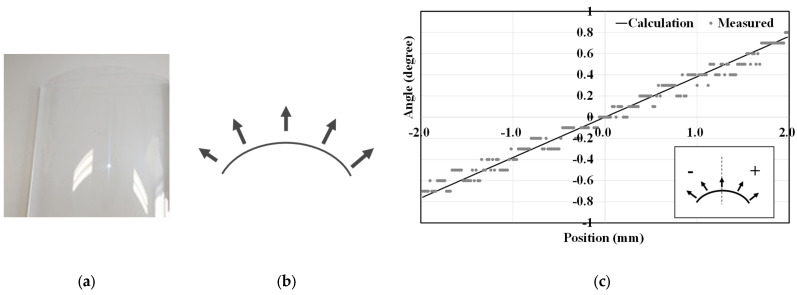
Verification of measurement accuracy II: curved acrylate sample. Surface normal vectors measured at each position along the x-axis using the proposed measurement apparatus. (**a**) Photograph of the curved acrylate sample. (**b**) Schematic diagram of the curved sample geometry. (**c**) Measured surface normal vector distribution.

The second verification was conducted using a curved sample. The sample was a curved acrylate plate with a known radius of curvature of 150 mm, as shown in Figure 12. For such a cylindrical surface, the surface normal vectors are expected to vary uniformly across the surface. Figure 12 shows the surface normal vectors measured at each position, which exhibit good agreement with the analytically calculated values. Taking the surface normals calculated from the reference curve as the ground truth, the mean error was 0.002° and the root mean square error (RMSE) in the x-direction was 0.064°.

To verify repeatability, the RMSE of the differences between consecutive measurements of the same sample was 0.035° in the x-direction and 0.044° in the y-direction. The sample used was Inkjet paper A, and the maximum RMSE among three consecutive measurements is reported. The measurement error and repeatability were both approximately 0.05°, which is mainly attributed to the quantization error associated with the 0.1° angular resolution.

## 4. Discussion

### 4.1. Differences from Conventional Technologies

The proposed method is based on reflectometry; however, it introduces a novel implementation strategy in which the illumination angle is controlled by laterally translating a small light source, rather than mechanically rotating the optical assembly. This approach significantly reduces system complexity and enables high-precision angular scanning that is difficult to achieve with conventional systems.

The objective of this study is to measure surface normals for analyzing the appearance of specular reflection. Accordingly, the measurement specifications were defined to be consistent with the capabilities of the human visual system. Specifically, the measurement area was set to 17 × 17 mm or larger, the spatial resolution to 0.1 mm or finer, the angular range to ±3.0°, and the angular resolution to 0.2° or better. Considering the trade-offs inherent in measurement techniques, conventional approaches are discussed under these requirements, including reflectometry, photometric stereo, confocal microscopy, and white-light interferometry.

Conventional reflectometry (i.e., gonio-photometric methods) provides highly accurate angular measurements but is inherently limited to point-wise acquisition. Generating collimated illumination over a 0.02 mm square area is challenging, and spatial scanning is time-consuming. We have previously investigated measurements using a 0.1 mm-diameter light source [14] and imaging with a rotating mirror [2]; however, for imaging-based measurements, the proposed variable-directional illumination method is more effective.

The proposed method is related to deflectometry based techniques, particularly Phase Measuring Deflectometry (PMD) [7]. However, it differs in that angular information is not inferred from pattern distortion. Furthermore, the proposed approach does not require complex calibration or precise pattern control, enabling a more robust and practical measurement framework.

Photometric stereo is a widely used technique for estimating surface normals, but it is primarily designed for diffuse surfaces. Although methods have been proposed for specular surfaces by incorporating pre-measured BRDF data, these approaches tend to be unstable.

In interferometric and confocal methods, surface normals are obtained indirectly by differentiating measured height maps, which amplifies noise and reduces robustness [16,17]. A key distinction lies in whether the measurement directly captures specular reflection characteristics or only surface geometry. Inferring specular reflection from surface height requires additional assumptions or auxiliary information, such as reflectance properties. In contrast, the proposed method directly utilizes specular reflection as the primary measurement signal. The acquired data correspond to images of specular reflected light under controlled illumination angles.

The proposed method satisfies the required specifications except for its limited angular range.

The robustness of the proposed method arises from exploiting the localized and peaked nature of specular reflection lobes. This enables accurate estimation of surface normals, particularly for materials with strong specular reflectance. The convex intensity distribution of specular reflection lobes in the BRDF thus provides a physically grounded basis for robust normal estimation. In practical applications, optical corrections and sensor noise must be considered. However, the proposed method offers advantages in this regard. Conventional camera-based measurements of reflected light distributions require complex corrections for illumination uniformity, beam collimation, and lens distortion. In contrast, the proposed method relies on detecting intensity peaks at each measurement location, which simplifies processing. At this level of specification, the optical transfer function (OTF) also contributes beneficially by smoothing noise and facilitating peak detection.

The apparatus further enhances robustness and ease of implementation. The variable-directional illumination strategy reduces system complexity, with the only moving component being a lightweight LED mounted on a standard xy-stage, rather than a goniometric stage. Since the optical system remains fixed, alignment errors are minimized. Moreover, the use of collimated illumination and a telecentric imaging system largely eliminates the need for precise focusing. Consequently, the proposed method is more robust and stable than conventional systems in both measurement principle and apparatus configuration.

By linking surface features to measurable optical responses from a physical optics perspective, this study provides a framework for objective analysis of material appearance. Furthermore, the proposed method may enable the estimation of surface height distributions.

### 4.2. Industrial Inspection Applications

**Figure 13:**
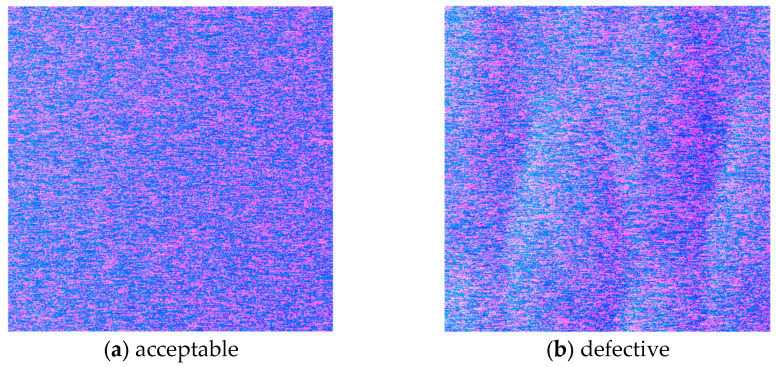
Normal maps of aluminum samples obtained during polishing using the proposed measurement apparatus. (**a**) acceptable and (**b**) defective. Defective regions exhibit distinctive patterns that are useful for identifying polishing issues.

Polishing is a common process in the manufacturing of industrial products, where surfaces are uniformly finished to achieve mirror-like characteristics. This process is widely applied to products such as semiconductor wafers and metal components. Figure 13 shows normal maps of acceptable and defective aluminum materials obtained during polishing, as measured using the proposed prototype measurement apparatus.

These surfaces are also inspected visually based on specular reflected light. In particular, the distinctive patterns that appear on defective products are useful for estimating the causes of problems in the polishing process. Inspection using normal maps makes these patterns easier to distinguish and enables them to be recorded quantitatively.

### 4.3. Applicable Surface Types and Limitations

The measurable angular range of the proposed system is approximately ±1.1°, which constrains the types of surfaces that can be analyzed. This limitation arises from the angular scanning range of the illumination system and the requirement to capture specular reflections within the field of view. Consequently, the method is best suited for surfaces that are locally near-planar and exhibit moderate to strong specular reflectance. Typical industrial examples include glossy printing media (e.g., high-gloss-coated and inkjet papers), polymer films, painted or coated surfaces, polished tiles, and metallic surfaces such as brushed or polished aluminum. In these applications, surface normal variations are typically small (within a few degrees), yet they strongly influence visual appearance, including gloss, texture, and non-uniformity. The proposed method is particularly effective in capturing such subtle variations due to its high angular sensitivity.

In contrast, the method is not suitable for surfaces with large slopes or complex geometries, where the surface normals fall outside the measurable angular range. Furthermore, for nearly ideal mirror-like surfaces (e.g., optical-grade mirrors or semiconductor wafers), where surface deviations are extremely small, interferometric methods may offer higher sensitivity for detecting nanoscale variations.

Therefore, the proposed method should be regarded as a complementary technique that targets the intermediate regime between visual inspection and high-precision optical metrology, particularly for industrial appearance evaluation.

### 4.4. Appearance Reproduction Using Computer Graphics

By utilizing the measured surface normal vector distributions, surface appearance can be reproduced using computer graphics techniques [18,19,20]. Because the proposed method employs specular reflection images as the source data, appearance reproduction under the measured conditions is straightforward. A key advantage of this approach lies in its ability to perform simulations under modified conditions. Photographic images provide visual information limited to a single set of illumination and viewing conditions. In contrast, the proposed framework enables simulation and quantitative comparison not only for changes in illumination and viewing directions, but also for variations in appearance resulting from modifications to the surface normal distribution. This capability allows systematic evaluation of appearance variations that cannot be directly captured by imaging alone. Consequently, the proposed method provides a more comprehensive understanding of how surface characteristics influence human visual perception and is expected to contribute to material design and surface engineering in industrial applications.

## 5. Conclusions

We developed a dedicated measurement apparatus capable of capturing surface normal vectors at 1024 × 1024 sampling points with a spatial resolution of 0.02 × 0.02 mm and an angular resolution of approximately 0.1°. The spatial distribution of the measured surface normal vectors reflects surface roughness, which directly influences perceived texture. The resulting high-resolution normal maps enable quantitative surface analysis and are applicable to industrial inspection. Through this approach, a more comprehensive understanding of the relationship between surface characteristics and human visual perception can be achieved. Furthermore, the proposed method provides valuable insights for material design and surface engineering in industrial applications.

## Data Availability

The data presented in this study are available on request from the corresponding author due to privacy.
